# A Case of Disseminated Histoplasmosis Detected in Peripheral Blood Smear Staining Revealing AIDS at Terminal Phase in a Female Patient from Cameroon

**DOI:** 10.1155/2012/215207

**Published:** 2012-11-29

**Authors:** Christine Mandengue Ebenye

**Affiliations:** Cliniques Universitaires des Montagnes, Bangangté, Cameroon

## Abstract

Histoplasmosis is endemic in the American continent and also in Sub-Saharan Africa, coexisting with the African histoplasmosis. Immunosuppressed patients, especially those with advanced HIV infection develop a severe disseminated histoplasmosis with fatal prognosis. The definitive diagnosis of disseminated histoplasmosis is based on the detection of *Histoplasma capsulatum *from patient' tissues samples or body fluids. Among the diagnostic tests peripheral blood smear staining is not commonly used. Nonetheless a few publications reveal that *Histoplasma capsulatum *has been discovered by chance using this method in HIV infected patients with chronic fever and hence revealed AIDS at the terminal phase. We report a new case detected in a Cameroonian woman without any previous history of HIV infection. Peripheral blood smear staining should be commonly used for the diagnosis of disseminated histoplasmosis in the Sub-Saharan Africa, where facilities for mycology laboratories are unavailable.

## 1. Introduction

Histoplasmosis is a systemic infection caused by the dimorphic fungus *Histoplasma capsulatum* (Hc). This infection is endemic in the American continent but also in Sub-Saharan Africa, coexisting with African histoplasmosis [1]. People with weakened immune system, especially those with advanced HIV infection, are at the greatest risk for developing severe and disseminated histoplasmosis (DH) with fatal prognosis [2, 3]. These cases commonly manifest clinical features such as high persistent fever, fatigue, and weight loss, accompanied with hepatosplenomegaly and lymphadenopathy, resembling closely disseminated tuberculosis. So the definitive diagnosis is based on detecting Hc from patient's tissue samples or body fluid [4]. A number of laboratory tests are available including peripheral blood smear staining. However this is not commonly used. A few studies reveal that Hc has been detected by chance using this method in HIV infected patients with chronic fever and hence revealed AIDS at the terminal phase [4–8]. In fact, the diagnosis of DH in HIV infected individuals is classified as AIDS since 1987 [2]. We report a new case diagnosed in a Cameroonian woman but with no previous history of HIV infection. We want to emphasize that in Sub-Saharan Africa, whenever clinicians consult a patient with high persistent fever, fatigue, and severe weight loss, they should always think of DH. 

## 2. Case Presentation

**Figure 1:**
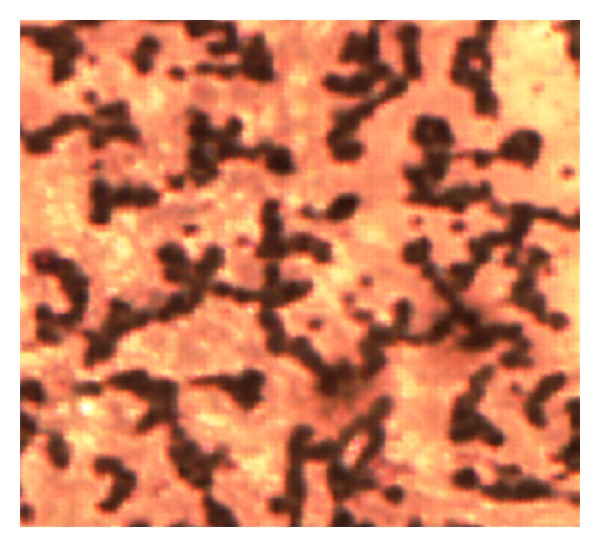
MGG peripheral blood film (×40); *Histoplasma capsulatum* scattered within red blood cells, with an eccentric chromatin and surrounded with a clear halo.

**Figure 2:**
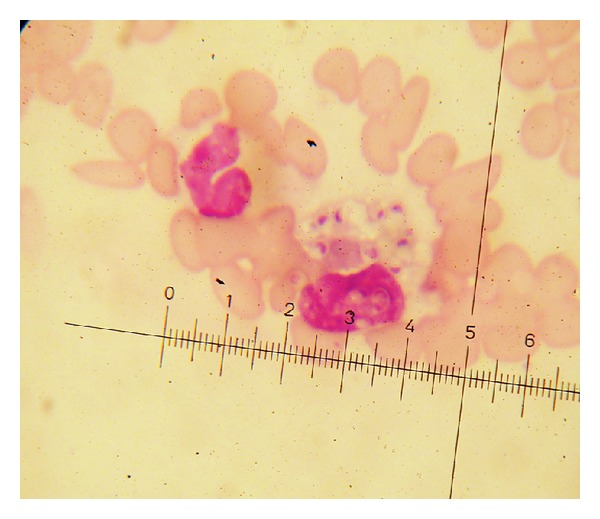
MGG peripheral blood film (×100); *Histoplasma capsulatum* inside and next to a monocyte.

**Figure 3:**
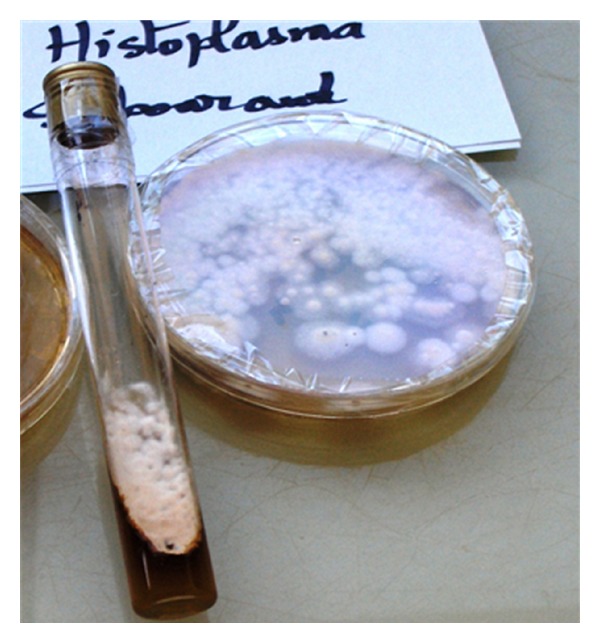
*Histoplasma capsulatum* colonies in Sabouraud medium.

A 25-year-old unemployed Cameroonian woman was admitted with one-month history of high persistent fever, weight loss, and fatigue. Her past history, especially concerning HIV infection, was unknown. She had been self-medicated with antipyretic and antimalaria drugs but she did not recover. On physical examination the patient appeared weak, with fatigue and conjunctival pallor. Her temperature was 38,5°C, with a pulse 30 beats per minute. Her blood pressure was 80/50 mmHg and she had severe weight loss of more than 10 kg. No lymphadenopathy was noted and findings on lung and cardiac examination were unremarkable. The patient's abdomen was distended and tender with a palpable liver tip. She had difficulties with attention and on neurological examination she could not walk because of fatigue but she showed no focal neurologic deficits. Electrocardiogram (ECG) and chest X-Ray were normal. Laboratory test results revealed pancytopenia, hemoglobin of 7.3 g/dL, and ASAT: 352 IU/L, ALAT: 59 IU/L. HIV_1_ serology was positive and CD_4_ was 7/mm^3^. Urine culture was sterile. The peripheral blood film showed no indication of malaria parasites, but it revealed the presence of yeast-like organisms with clear halo and eccentric chromatin, 2 to 4 *μ*m in diameter, inside and next to monocytes and also scattered within red blood cells (Figures 1 and 2), confirming to the morphology of *Histoplasma capsulatum* var *capsulatum*. Blood culture in Sabouraud medium isolated Hc (Figure 3). The patient died just after all the samples were collected. The postmortem diagnosis of DH revealing AIDS in terminal phase was concluded.

## 3. Discussion 

Many specific methods for diagnosing DH in AIDS patients are available. 

Antigen detection appears to be the most sensitive rapid assay, which detects Hc in 86–90% of AIDS patients [3, 4, 9]. However false-positive errors can occur in patients with blastomycosis or paracoccidioidomycosis [9]. 

PCR assay is also a rapid, sensitive, and specific method for diagnosing DH but not yet for routine use [9–11]. 

Direct microscopic examination of specimens such as bronchial aspirates, bone marrow, biopsy, or peripheral blood smear (notably the buffy coat), after staining with Giemsa, May-Grünwald Giemsa (MGG), Wright, periodic acid of Schiff (PAS), or Gomori methenamine silver, is the simplest, rapid, least expensive but relatively contributive test with sensitivity of less than 50% [12]. *Histoplasma capsulatum* var *capsulatum* (Hcc) appears as intracellular tiny round or oval bodies from 1–4 *μ*m in diameter, with a recognizable clear halo surrounding a central or eccentric stained chromatin but can be mistaken for *Candida glabrata*, *Penicillium marneffei*, *Pneumocystis* (carinii) *jiroveci*, *Toxoplasma gondii*, *Leishmania donovani*, staining artifact, and *Cryptococcus neoformans* [9, 12]. 

Culture is risky because of contamination but remains a gold standard for diagnosing DH with 100% specificity, sensitivity depending on the fungal loads, and the experience of the laboratory [7, 8, 12]. Blood cultures using lysis-centrifugation technique or instrumented blood culture techniques such as BACTEC or Becton Dickinson are more contributive than standard culture (Sabouraud or agar medium) [9, 12]. However in either standard or special techniques, 35–37°C three to six weeks are required for growth and identification, with treatment initiation. The fungal colony appears initially smooth but becomes cottony and brownish with age. Microscopically, they are composed of septated hyphae with micro- and macroconidia in various developmental stages. 

In Sub-Saharan African countries most of these above methods for diagnosing DH still remain very expensive procedures and therefore they are unavailable. Direct examinations of specimens after staining are available, but peripheral blood smear staining is not yet commonly used. In Cameroon few cases of DH diagnosed in AIDS patients were detected on skin biopsies after staining with Gomori methenamine and culture in Sabouraud medium [13]. The reported case demonstrates DH detected by chance in peripheral blood smear revealing AIDS at the terminal stage in a Cameroonian woman with no previous history of HIV infection. Up until now this is the first case reported.

Such cases are underestimated in the tropics. In fact fever is one of the features of DH which may be mistaken for any tropical endemic infection such as malaria, tuberculosis or HIV infection. And also, in Sub-Saharan Africa, histoplasmosis is still misunderstood because of similar clinical findings with tuberculosis [5, 13]. Besides, the lacks of health facilities and the low financial conditions expose individuals to self-medication in case of fever, without any detection of the disease. Histoplasmosis is misdiagnosed and its prevalence is unknown because of the lack of facilities for mycology laboratories. These situations increase the risk of death, underestimate cases of DH and AIDS at the terminal stage, and, so, delay the setup of efficient rules of fighting against this emerging infectious disease. Hence, it seems mandatory for the clinicians, in Sub-Saharan Africa to systematically rule out DH, malaria, tuberculosis, and HIV infection in case of persistent fever associated with severe weight loss. 

In that case peripheral blood smear staining, a simplest and least expensive procedure may be helpful. 

In conclusion, peripheral blood smear should be commonly used in Sub-Saharan Africa where facilities for mycology laboratories are unavailable. Close collaboration between clinicians and biologists would prove beneficial.
